# Regular sleep patterns, not just duration, critical for mental health: association of accelerometer-derived sleep regularity with incident depression and anxiety

**DOI:** 10.1017/S0033291725101281

**Published:** 2025-08-15

**Authors:** Dong-Run Li, Zheng-Xuan Li, Ming-Hui Li, Bang-Quan Liu, Qian Fang, Jia-Cheng Liu, Wen-Rui Zheng, Ting-Ting Gong, Shan-Yan Gao, Qi-Jun Wu

**Affiliations:** 1 Department of Clinical Epidemiology, Shengjing Hospital of China Medical University, Shenyang, China; 2 Department of Epidemiology, School of Public Health, China Medical University, Shenyang, China; 3 Department of Obstetrics and Gynecology, Shengjing Hospital of China Medical University, Shenyang, China; 4 Clinical Trials and Translation Center, Shengjing Hospital of China Medical University, Shenyang, China; 5 NHC Key Laboratory of Advanced Reproductive Medicine and Fertility (China Medical University), National Health Commission, Shenyang, China

**Keywords:** anxiety, depression, sleep duration, sleep regularity

## Abstract

**Background:**

Depression and anxiety are prevalent mental health disorders. While sleep duration has been extensively studied, sleep regularity may play a critical role. We aimed to examine associations between objectively measured sleep regularity and incident depression and anxiety and to investigate whether meeting recommended sleep duration modifies these associations.

**Methods:**

In 79,666 UK Biobank participants without baseline depression or anxiety, wrist accelerometers worn for 7 days yielded a sleep regularity index (SRI) and average sleep duration. SRI was categorized as irregular (≤51), moderately irregular (52–70), or regular (≥71). Sleep duration was classified by age-specific recommendations (7–9 hours for ages 18–64 years; 7–8 hours for over 65 years). Cox regression models assessed associations between sleep parameters and mental health outcomes.

**Results:**

During a median follow-up of 7.5 years, 1,646 participants developed depression, and 2,097 developed anxiety. Compared to irregular sleepers, regular sleepers had a 38% lower depression risk (hazard ratio [HR], 0.62; 95% confidence interval [CI], 0.52–0.73) and a 33% lower anxiety risk (HR, 0.67; 95%CI, 0.58–0.77). Participants with both irregular sleep and nonrecommended duration exhibited the highest risks (depression HR, 1.91; 95%CI, 1.55–2.35; anxiety HR, 1.61; 95%CI, 1.35–1.93). Notably, irregular sleepers who met duration guidelines still faced elevated risks (depression HR, 1.48; 95%CI, 1.18–1.86; anxiety HR, 1.35; 95%CI, 1.11–1.64).

**Conclusions:**

Greater sleep regularity is independently associated with lower depression and anxiety risk regardless of sleep duration, suggesting that sleep–wake consistency should be considered in mental health promotion strategies alongside traditional sleep duration recommendations.

## Introduction

Mental health disorders, particularly depression and anxiety, represent a significant global health burden with increasing prevalence rates worldwide (COVID-19 Mental Disorders Collaborators, 2021). Depression affects approximately 280 million people globally, while anxiety disorders affect an estimated 301 million individuals, with both conditions often co-occurring and sharing common risk factors (GBD, 2019). Given their substantial societal impact, identifying modifiable risk factors for these disorders remains a public health priority.

Sleep plays a fundamental role in maintaining mental health and emotional regulation. Extensive research has established robust associations between various sleep parameters and mental health outcomes (Scott et al., 2021; Sun et al., 2022). While numerous studies have focused on sleep duration, with both short and long sleep durations associated with adverse mental health outcomes (Chunnan, Shaomei, & Wannian, 2022), mounting evidence suggests that other dimensions of sleep may be equally important.

Sleep regularity, the day-to-day consistency in sleep–wake timing rather than the amount of sleep obtained, has emerged as a potentially critical component of healthy sleep (Windred et al., 2024). The sleep regularity index (SRI), developed by Phillips et al., quantifies the probability of an individual being in the same sleep–wake state at any two time points 24 hours apart (Phillips et al., 2017), thus capturing the stability of circadian timing independent of total sleep duration. The biological underpinnings of sleep regularity are closely tied to the circadian system (Charrier, Bertrand, Pierre, & Sylvie, 2017). Disruption of these rhythms, often manifested as irregular sleep patterns, may trigger cascading effects on neuroendocrine function, inflammatory processes, and neurotransmitter systems implicated in mood regulation (Logan & McClung, 2019; Walker, Walton, DeVries, & Nelson, 2020).

Despite growing recognition of the importance of circadian rhythm, current research on sleep and mental health has several limitations. Studies have predominantly focused on sleep duration and quality, with insufficient attention to sleep regularity (Matricciani et al., 2018). Most existing studies rely on self-reported sleep measures, which are subject to recall bias and limited in capturing day-to-day variations in sleep patterns (Cespedes et al., 2016; Lauderdale et al., 2008). Additionally, large-scale prospective studies using objective sleep measurements to examine the relationship between sleep regularity and incident depression or anxiety are notably scarce.

Therefore, in this study, based on the UK Biobank database, we aimed to investigate the associations between objectively measured sleep regularity, sleep duration, and risk of developing depression and anxiety, and determine whether achieving recommended sleep duration mitigated the effects of irregular sleep on depression and anxiety incidence.

## Methods

### Study design

The data used in this study were derived from the UK Biobank, a large, population-based prospective cohort study that included over 500,000 participants aged 38–73 years, recruited between 2006 and 2010 (Allen et al., 2024). Participants completed an initial assessment at the time of recruitment, designed to capture information across a broad range of health and lifestyle factors, through questionnaires and physical measurements (Sudlow et al., 2015). Between February 2013 and December 2015, a total of 103,683 participants with valid e-mail addresses from the UK Biobank were randomly invited to participate in a 7-day wrist-worn accelerometer study. After excluding participants with depression or anxiety at baseline, a total of 89,533 participants were included. Further exclusion was conducted for participants with unreliable accelerometer data or missing data required for calculating the SRI and sleep duration. Ultimately, a total of 79,666 participants were included in the final analysis (Supplementary Figure 1).

All participants gave written consent, and all data collection was conducted following the Declaration of Helsinki. The study was approved by the North West Multicenter Research Ethics Committee in the United Kingdom (Allen et al., 2024) and was conducted under UK Biobank application number 211772.

### Sleep regularity and sleep duration

From this baseline cohort, participants wore Axivity AX3 devices (Axivity, Newcastle upon Tyne, UK) on their dominant wrist for 7 days under free-living conditions. The Axivity AX3 accelerometer is a lightweight, wrist-worn device designed to capture triaxial acceleration data continuously at a high resolution of 100 Hz, with a dynamic range of ±8 gravity. The raw accelerometer data were preprocessed using the Euclidean Norm Minus One method, which removes the gravitational constant from the vector magnitude of the triaxial signals and expresses the resulting movement in milligravity units. Sleep status (awake or asleep) at any given time was estimated using the open-source R package GGIR, version 2.7-1, which leverages established algorithms (Vincent T. van Hees et al., 2015; Vincent Theodoor van Hees et al., 2018). In the absence of sleep diary data, GGIR uses a specific algorithm to define a daily sleep period time window for each participant (Vincent Theodoor van Hees et al., 2018). This window represents the timeframe between the start and end of the main daily sleep episode, during which sustained inactivity, arm angle changes consistent with lying position, and the absence of intermittent movement patterns typical of conscious rest are interpreted as sleep. The validity of accelerometer-based sleep assessment has been established through comparison with polysomnography. The GGIR algorithm shows 83% accuracy in sleep–wake classification, with the specific Axivity AX3 device demonstrating high sensitivity and specificity for sleep detection (Doherty et al., 2017; van Hees et al., 2018). By default, the algorithm does not detect sleep episodes occurring outside this defined window and thus cannot identify naps.

Sleep regularity was evaluated using the SRI, a metric that quantifies the consistency of daily sleep patterns by comparing consecutive days (Phillips et al., 2017). The SRI calculates the average concordance in sleep–wake states across all time pairs separated by 24 hours. For example, two individuals may both achieve 8 hours of sleep, but one who consistently sleeps from 10 PM to 6 AM would have a high SRI score, while another who varies their sleep timing daily (e.g., 10 PM–6 AM on Monday, 2 AM–10 AM on Tuesday) would have a low SRI score despite similar sleep duration. Participants were included in the SRI calculation only if they had at least five valid days of accelerometer data, recorded for a minimum of 16 hours per day. This ensured that only high-quality data were used for downstream analyses. An SRI score of 100 indicates perfectly consistent sleep–wake patterns, while a score of 0 reflects completely random patterns. Participants were categorized as irregular (0–25th percentile, SRI ≤ 51); moderately irregular (25–75th percentile, 52 ≤ SRI ≤ 70); and regular (75–100th percentile, SRI ≥ 71) sleepers (Windred et al., 2021).

Sleep duration data were extracted based on the same study days used for calculating the SRI. For each participant, the daily average sleep duration was calculated based on the period of sustained inactivity between sleep onset and offset times, as estimated by GGIR (Windred et al., 2024). The overall average sleep duration for each participant was then derived by aggregating these daily values across the study period. Participants were categorized based on whether they met the sleep duration recommendation of 7–9 hours per day for adults aged 18–64 years or 7–8 hours per day for adults aged over 65 years (Hirshkowitz et al., 2015; Ross et al., 2020).

### Outcomes

Depression and anxiety diagnoses were identified in participants through multiple sources, including hospital inpatient records, primary care, and death registry data. The primary care data captured information recorded by healthcare professionals in general practice settings, which typically identifies mental health conditions earlier than hospital admissions. The classification of depressive and anxiety disorders was based on the 10th Revision of the International Classification of Diseases, using diagnostic codes F32 (depressive episode) and F33 (recurrent depressive disorder) for depression, and F40–F48 (neurotic, stress-related, and somatoform disorders) for anxiety, including all subcategories (Fan et al., 2025). Follow-up was conducted until depression or anxiety diagnosis, death, or the end of follow-up (October 31, 2022 for England, May 31, 2022 for Wales, and August 31, 2022 for Scotland), whichever came first.

### Assessment of covariates

Covariates included in the analysis were selected based on their potential confounding effects on the association between sleep regularity and health outcomes (Chaput et al., 2024; Yang et al., 2023). Baseline characteristics included age at recruitment (continuous, years), sex (male or female), body mass index (BMI, continuous), and ethnicity (White or non-White). Health-related covariates encompassed smoking status (never, previous, current, or unknown/missing); alcohol consumption status (never, previous, current, or unknown/missing); physical activity level (low: <600, moderate: 600–3000, and high: >3000 metabolic equivalent tasks minutes/week); healthy diet score (continuous); and shift work (yes, no, or unknown/missing). The healthy diet score was calculated based on the consumption of fruits, vegetables, processed meat, red meat, fish, whole grains, and refined grains (Supplementary Table 1). A score of 4 or above was considered indicative of a high diet quality. Socioeconomic factors included household income (<£31,000, ≥£31,000, or unknown/missing); education level (college level, below college level, no qualification, or unknown/missing); and Townsend deprivation index (continuous). Additionally, the season during which the accelerometer was worn (spring, summer, autumn, or winter) was included as a covariate. Detailed coding information in the UK Biobank is shown in Supplementary Table 2.

### Statistical analysis

Baseline characteristics of the participants were described as means (standard deviation) or medians (interquartile range) for continuous variables and proportions for categorical variables. In the statistical analysis, chi-square tests were used for categorical variables. For continuous variables, a range of tests were applied, including analysis of variance, Wilcoxon tests, or Kruskal–Wallis tests, depending on the data distribution and study requirements. Furthermore, we compared baseline characteristics between participants included and those excluded from the final analysis. Pearson’s correlation coefficient was calculated to quantify the linear association between two sleep parameters.

The proportionality assumption of Cox regression models was verified through Schoenfeld residuals analysis, confirming compliance with proportional hazards criteria across all variables (*P* > 0.05). Multivariable-adjusted Cox regression analyses were performed to assess the associations between SRI and sleep duration with risks of depression and anxiety, with results reported as hazard ratios (HRs) and 95% confidence intervals (CIs). SRI was analyzed both as continuous (per standard deviation increase) and categorical variables (irregular: 0–25th percentile, moderately irregular: 25–75th percentile, regular: 75–100th percentile). All models adopted the time since accelerometer deployment as the temporal scale. Two models were constructed. Model 1 adjusted for age at recruitment, sex, BMI, and ethnicity. Model 2 further adjusted for annual household income, education level, smoking status, Townsend deprivation index, alcohol consumption status, physical activity level, healthy diet score, shift work, the season of accelerometer wear, and sleep duration. Furthermore, to explore potential nonlinear relationships between sleep patterns and mental health outcomes, we applied restricted cubic spline (RCS) models with four knots (at the 5th, 35th, 65th, and 95th percentiles), adjusting for all covariates included in Model 2.

To comprehensively examine the potential interaction and combined effects of SRI and meeting the recommended sleep duration on the risk of anxiety and depression, a joint analysis was conducted. Participants were categorized into six distinct groups based on combinations of SRI levels and sleep duration categories, with the group characterized by regular sleep patterns and meeting the recommended sleep duration as the reference. Stratified analyses were performed to evaluate the independent effects of sleep regularity on anxiety and depression risk within subgroups defined by sleep duration. The multiplicative interaction between SRI and sleep duration was evaluated using the “interactionR” package in R. In the joint and stratified analyses, sleep duration was treated as a grouping variable rather than a covariate to avoid overadjustment.

Subgroup analyses were conducted to determine whether the associations varied across specific demographic and lifestyle factors. These factors were guided by previous literature, including age at recruitment, sex, BMI, education level, annual household income, Townsend deprivation index, diet quality, and physical activity level (Kandola et al., 2021; Dankang et al., 2023; Lv et al., 2024; Xu, Yin, & Gong, 2023). To ensure the robustness and reliability of the primary findings, several sensitivity analyses were conducted: (1) excluding participants diagnosed with depression or anxiety within the first 2, 5, and 7 years of follow-up to reduce potential reverse causation bias (Lu et al., 2024); (2) removing individuals with missing or incomplete covariate data; (3) further adjusted for: polygenic risk scores for depression and anxiety (Supplementary Method 1), region, chronotype (morning-type or evening-type preference), neuroticism scores from the revised Eysenck Personality Questionnaire (Lyall et al., 2018), history of chronic diseases at baseline, including hypertension, diabetes, and hyperlipidemia (Cribb et al., 2023); (4) redefining depression and anxiety cases using Patient Health Questionnaire (PHQ)-9 and Generalized Anxiety Disorder (GAD)-7 questionnaires administered between 2016 and 2017 to avoid bias from relying solely on medical records. A PHQ-9 or GAD-7 total score of ≥10 was considered depression or anxiety symptoms (Supplementary Method 2) (Fan et al., 2025); and (5) stricter baseline exclusion criteria by excluding participants with subclinical symptoms (PHQ-4 assessment positive) or any psychotropic medication use at baseline.

## Results

### Participant characteristics

This study included a total of 79,666 participants, with an average follow-up time of 7.5 years (interquartile range: 6.98–8.05 years). During the follow-up period, 1,646 (2.07%) participants were diagnosed with depression, and 2,097 (2.63%) were diagnosed with anxiety. The baseline demographic and clinical characteristics of participants, stratified by sleep regularity, are presented in Table 1. Compared with participants with irregular sleep patterns, those with regular sleep patterns were younger, had a higher proportion of females, and exhibited a lower BMI. Participants with regular sleep patterns also reported higher income levels and lower proportions of current smoking and alcohol consumption. Furthermore, they were more likely to engage in moderate to high levels of physical activity and follow a healthy dietary pattern. In contrast, participants with irregular sleep patterns were more likely to be employed in shift work. Comparison of included versus excluded participants revealed modest differences in baseline characteristics (Supplementary Table 3).

**Table 1.** Characteristics of participants at baseline

Characteristics	Total	Sleep regularity index		
		Irregular (Q_1_)	Moderate Irregular (Q_2_–Q_4_)	Regular (Q_5_)
Number of population	79,666	15,934	47,799	15,933
Age, mean (SD), year	61.53 (7.87)	62.26 (7.78)	61.47 (7.85)	60.98 (7.94)
BMI, mean (SD), kg/m^2^	26.62 (4.43)	28.02 (4.95)	26.52 (4.28)	25.51 (3.96)
Sleep duration, mean (SD), hours	7.22 (1.00)	6.94 (1.24)	7.26 (0.95)	7.36 (0.81)
Townsend deprivation index, mean (SD)	−1.77 (2.79)	−1.28 (3.05)	−1.83 (2.75)	−2.06 (2.60)
Sex				
Female	43,620 (54.75)	6,188 (38.84)	26,292 (55.01)	11,140 (69.92)
Male	36,046 (45.25)	9,746 (61.16)	21,507 (44.99)	4,793 (30.08)
Ethnic background				
White	77,124 (96.81)	15,126 (94.93)	46,370 (97.00)	15,628 (98.09)
Non-White (mixed, Asian, Black, Chinese, and other ethnic groups)	2,542 (3.19)	808 (5.07)	1,429 (3.00)	305 (1.91)
Annual household income, £				
<31,000	26,921 (33.79)	6,388 (40.09)	15,604 (32.65)	4,929 (30.94)
≥31,000	44,499 (55.86)	7,960 (49.96)	27,315 (57.15)	9,224 (57.89)
Unknown/missing	8,246 (10.35)	1,586 (9.95)	4,880 (10.20)	1,780 (11.17)
Education status				
High	34,475 (43.27)	6,436 (40.39)	20,927 (43.78)	7,112 (44.64)
Middle	37,803 (47.45)	7,604 (47.72)	22,580 (47.24)	7,619 (47.82)
No above	6,592 (8.28)	1,694 (10.63)	3,834 (8.02)	1,064 (6.68)
Unknown/missing	796 (1.00)	200 (1.26)	458 (0.96)	138 (0.86)
Smoking status				
Never	45,905 (57.62)	8,244 (51.74)	27,774 (58.11)	9,887 (62.05)
Current	5,147 (6.46)	1,581 (9.92)	2,879 (6.02)	687 (4.31)
Previous	28,401 (35.65)	6,058 (38.02)	17,016 (35.60)	5,327 (33.43)
Unknown/missing	213 (0.27)	51 (0.32)	130 (0.27)	32 (0.21)
Alcohol consumption status				
Never	2,265 (2.84)	538 (3.38)	1,279 (2.68)	448 (2.81)
Current	75,393 (94.64)	14,841 (93.14)	45,409 (95.00)	15,143 (95.03)
Previous	1,935 (2.43)	537 (3.37)	1,065 (2.22)	333 (2.10)
Unknown/missing	73 (0.09)	18 (0.11)	46 (0.10)	9 (0.06)
Physical activity level^a^				
Low	11,011 (13.82)	2,607 (16.36)	6,389 (13,37)	2,015 (12.65)
Moderate	49,306 (61.89)	9,625 (60.41)	29,830 (62.41)	9,851 (61.83)
High	19,349 (24.29)	3,702 (23.23)	11,580 (24.22)	4,067 (25.52)
Diet quality^b^				
Healthy	53,281 (66.88)	9,683 (60.77)	32,145 (67.25)	11,453 (71.88)
Unhealthy	26,385 (33.12)	6,251 (39.23)	15,654 (32.75)	4,480 (28.12)
Employment shift				
No	42,915 (53.87)	7,628 (47.87)	26,289 (55.00)	8,998 (56.47)
Yes	6,289 (7.89)	1,733 (10.88)	3,597 (7.53)	959 (6.02)
Unknown/missing	30,462 (38.24)	6,573 (41.25)	17,913 (37.47)	5,976 (37.51)
Season of accelerometer wear				
Spring	18,059 (22.67)	3,509 (22.02)	10,897 (22.80)	3,653 (22.93)
Summer	20,688 (25.97)	3,694 (23.18)	12,469 (26.09)	4,525 (28.40)
Autumn	23,814 (29.89)	4,816 (30.23)	14,288 (29.89)	4,710 (29.56)
Winter	17,105 (21.47)	3,915 (24.57)	10,145 (21.22)	3,045 (19.11)

*Note:* Data are presented as mean (standard deviation) for continuous variables and *n* (%) for categorical variables.

*Abbreviations:* BMI, ‘body mass index’; MET, ‘metabolic equivalent tasks’; Q, ‘quantile’; SD, ‘standard deviation’.

a Physical activity was divided into three levels according to metabolic equivalent tasks (MET): low (<600 MET minutes/week), medium (600–3,000 MET minutes/week), and high (>3,000 MET minutes/week).

b Diet score was calculated based on the consumption of fruits, vegetables, processed meat, red meat, fish, whole grains, and refined grains. The diet score ranged from 0 to 7, with higher scores reflecting better diet quality, and ≥4 was considered a healthy diet.

Additionally, we examined the correlation between SRI and sleep duration. The weak correlation (r = 0.16, *P* < 0.001) suggests that these two sleep parameters capture distinct dimensions of sleep health.

### SRI with anxiety and depression risk

**Figure 1.**
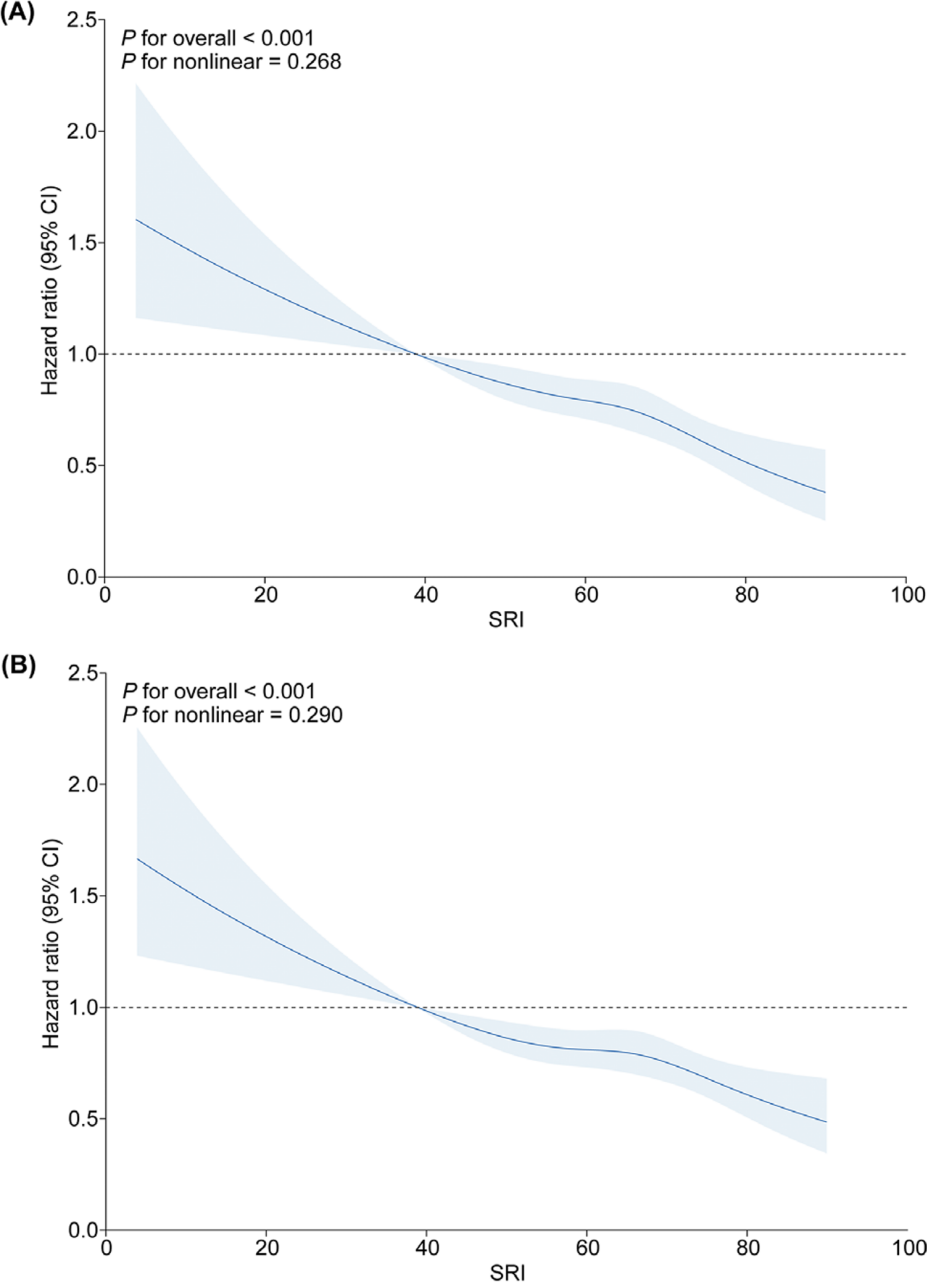
Dose–response relationship between SRI and depression (a) and anxiety (b) risk by RCS analysis. *Note*: CI, ‘confidence interval’; RCS, ‘restricted cubic spline’; SRI, ‘sleep regularity index’. Models were adjusted for age at recruitment, sex, body mass index, ethnicity, annual household income, education level, smoking status, Townsend deprivation index, alcohol consumption status, physical activity level, healthy diet score, employment shift, and season of accelerometer wear.

The associations between SRI and risks of anxiety and depression are presented in Table 2. In the fully adjusted models, each standard deviation increase in SRI was associated with a 14% decreased risk of depression (95% CI: 0.82, 0.90) and a 12% decreased risk of anxiety (95% CI: 0.85, 0.92). The RCS curve demonstrated a significant linear dose–response relationship between SRI and risk of depression and anxiety, as shown in Figure 1. When SRI scores were categorized, individuals with moderate irregular sleep patterns (HR: 0.80; 95% CI: 0.71, 0.90 for depression; HR: 0.82; 95% CI: 0.74, 0.91 for anxiety) and regular sleep patterns (HR: 0.62; 95% CI: 0.52, 0.73 for depression; HR: 0.67; 95% CI: 0.58, 0.77 for anxiety) had significantly lower risks of both disorders compared with the irregular group. The association between SRI and mental health outcomes persisted after adjusting for sleep duration, confirming that sleep regularity represents an independent risk factor beyond sleep quantity alone.

**Table 2.** Association between SRI and risk of depression and anxiety

SRI	Cases/total	HR (95% CI)	
		Model 1^a^	Model 2^b^
Depression			
Irregular (Q_1_)	434/15,934	1.00 (Ref.)	1.00 (Ref.)
Moderate irregular (Q_2_–Q_4_)	965/47,799	0.73 (0.65–0.82)	0.80 (0.71–0.90)
Regular (Q_5_)	247/15,933	0.55 (0.47–0.64)	0.62 (0.52–0.73)
*P* for trend		< 0.001	< 0.001
Per SD increment		0.81 (0.78–0.85)	0.86 (0.82–0.90)
Anxiety			
Irregular (Q_1_)	504/15,934	1.00 (Ref.)	1.00 (Ref.)
Moderate irregular (Q_2_–Q_4_)	1,242/47,799	0.77 (0.69–0.85)	0.82 (0.74–0.91)
Regular (Q_5_)	351/15,933	0.62 (0.53–0.71)	0.67 (0.58–0.77)
*P* for trend		< 0.001	< 0.001
Per SD increment		0.85 (0.82–0.89)	0.88 (0.85–0.92)

*Abbreviations*: CI, ‘confidence interval’; HR, ‘hazard ratio’; Q, ‘quantile’; Ref, ‘reference’; SD, ‘standard deviation’; SRI, ‘sleep regularity’ index.

a Model 1 was adjusted for age at recruitment, sex, body mass index, and ethnicity.

b Model 2 was further adjusted for annual household income, education level, smoking status, Townsend deprivation index, alcohol consumption status, physical activity level, healthy diet score, employment shift, season of accelerometer wear, and sleep duration based on model 1.

The results of subgroup analyses consistently supported the primary findings (Supplementary Tables 4 and 5). Additionally, significant multiplicative interactions were observed between the Townsend deprivation index and SRI on anxiety risk and between age and SRI on depression risk (all *P* for interaction <0.001). The observed associations remained robust in all sensitivity analyses (Supplementary Tables 6–10).

### Stratified analysis of SRI with depression and anxiety risks by sleep duration

**Figure 2.**
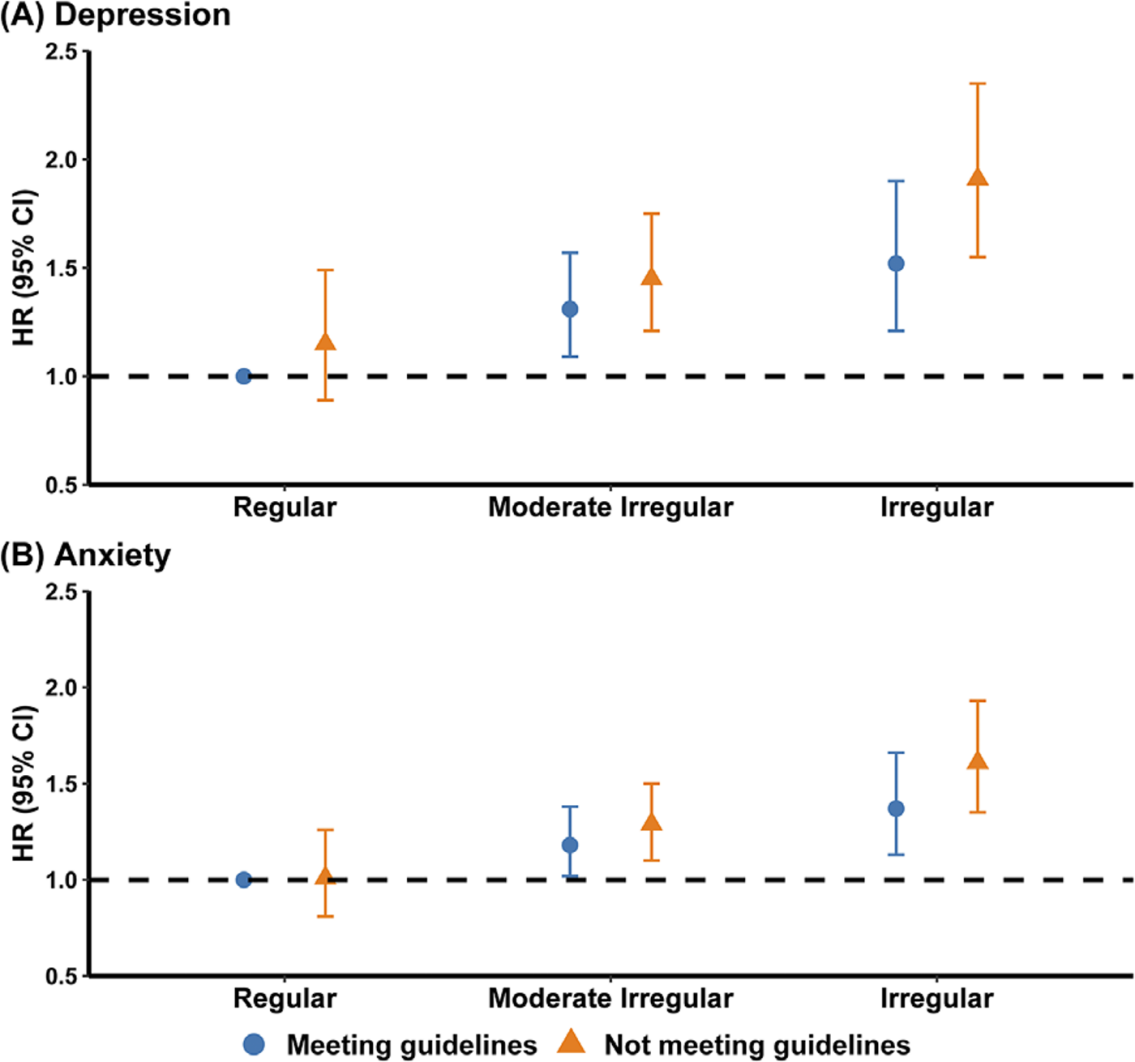
Joint associations of SRI and sleep duration with the risks of depression and anxiety. *Note*: CI, ‘confidence interval’; HR, ‘hazard ratio’; SRI, ‘sleep regularity index’. Meeting guidelines was defined as 7–9 hours daily sleep for adults aged 18–64 years or 7–8 hours for those ≥65 years, based on public health recommendations. Not meeting guidelines was classified as sleep durations outside these ranges. Models were adjusted for age at recruitment, sex, body mass index, ethnicity, annual household income, education level, smoking status, Townsend deprivation index, alcohol consumption status, physical activity level, healthy diet score, employment shift, and season of accelerometer wear.

Figure 2 illustrates the joint effects of sleep regularity and duration. Compared with participants who had regular sleep patterns and met duration guidelines, those with both irregular sleep and nonrecommended duration had a 91% higher risk of depression (95% CI: 1.55, 2.35) and a 61% higher risk of anxiety (95% CI: 1.35, 1.93) (Supplementary Tables 11 and 12).

**Figure 3.**
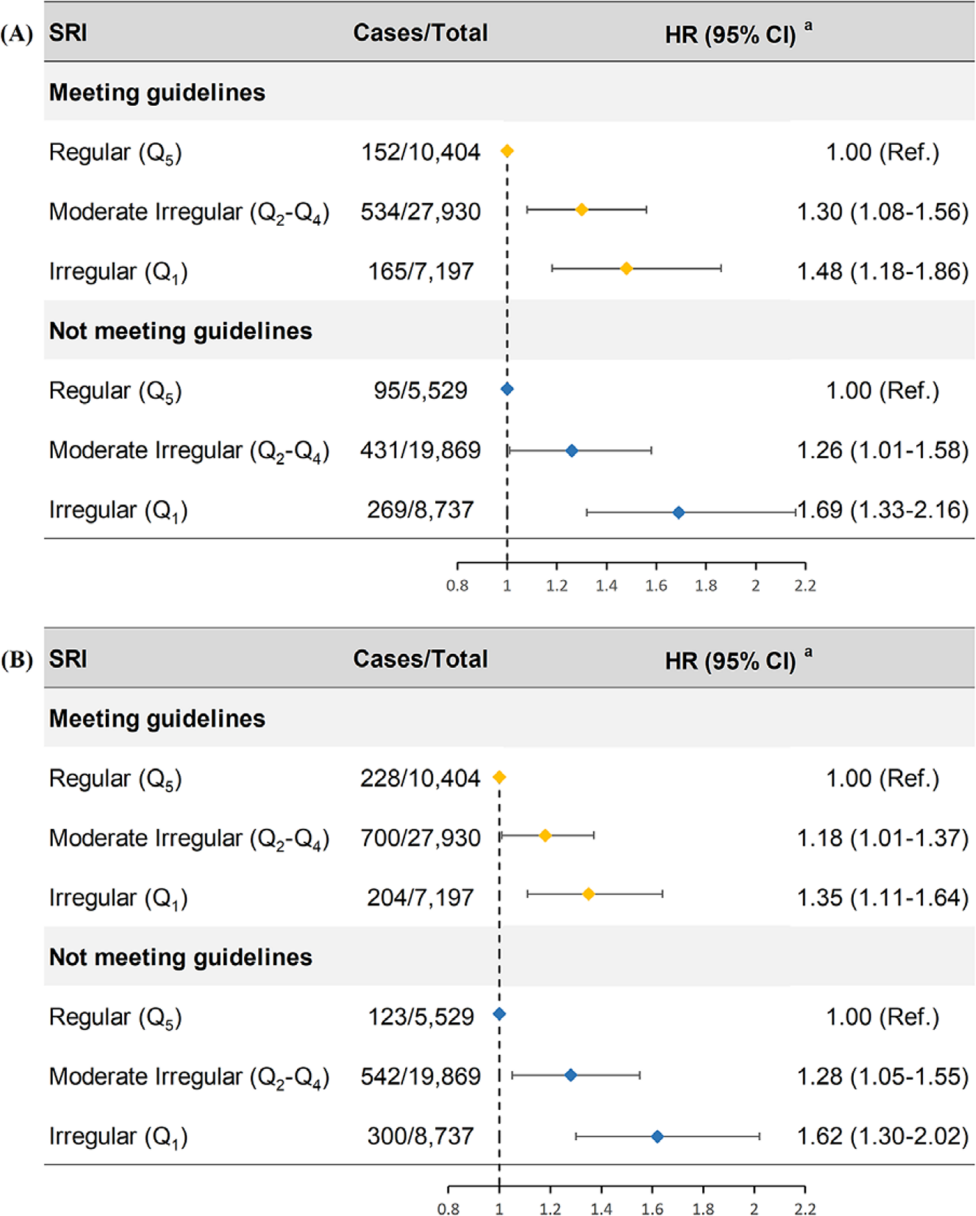
Association between SRI and risk of depression (a) and anxiety (b) according to sleep duration stratification. *Note*: CI, ‘confidence interval’; HR, ‘hazard ratio’; Q, ‘quantile’; Ref, ‘reference’; SRI, ‘sleep regularity index’. Meeting guidelines was defined as 7–9 hours daily sleep for adults aged 18–64 years or 7–8 hours for those ≥65 years, based on public health recommendations. Not meeting guidelines was classified as sleep durations outside these ranges. ^a^Models were adjusted for age at recruitment, sex, body mass index, ethnicity, annual household income, education level, smoking status, Townsend deprivation index, alcohol consumption status, physical activity level, healthy diet score, employment shift, and season of accelerometer wear.

In stratified analyses (Figure 3), irregular sleep was linked to greater depression and anxiety risks regardless of whether participants met duration recommendations. Even among those who achieved the recommended sleep duration, individuals in the lowest SRI category had a 48% higher risk of depression (95% CI: 1.18, 1.86) and a 35% higher risk of anxiety (95% CI: 1.11, 1.64) compared with regular sleepers. No significant interaction between SRI and sleep duration was observed (Supplementary Tables 13 and 14).

## Discussion

In this large prospective cohort study with objective sleep measurements, SRI was associated with an increased risk of depression and anxiety, with a significant linear dose–response relationship. Our joint analysis revealed a clear gradient of risk, with participants experiencing both irregular sleep patterns and adverse sleep duration exhibiting the highest risk of developing these disorders. Notably, even among participants meeting recommended sleep duration guidelines, those with irregular sleep patterns still exhibited significantly elevated risks of both conditions compared with the regular sleep group.

These results align with and expand upon recent investigations into sleep regularity as an emerging health indicator. Lyall et al. first linked disrupted circadian rhythmicity with mental health through a cross-sectional study (Lyall et al., 2018). Cribb et al. demonstrated that irregular sleep–wake patterns were associated with higher mortality risk in the UK Biobank cohort, with HR exhibiting a nonlinear dose–response relationship (Cribb et al., 2023). Similarly, Chaput et al. found that moderate and high sleep irregularity were associated with an increased risk of type 2 diabetes, even among participants who achieved the recommended sleep duration (Chaput et al., 2024). Our findings regarding mental health outcomes complement these studies on physical health, highlighting sleep regularity as a critical dimension beyond traditional sleep parameters. The observed linear relationship between SRI and a decreased risk of depression and anxiety suggests that even modest improvements in sleep regularity might yield mental health benefits, underscoring the importance of consistent sleep–wake patterns.

Additionally, our subgroup and interaction analyses found that regular sleep was more strongly associated with a reduced risk of depression among participants younger than 60 years, and with a lower risk of anxiety among those from socioeconomically disadvantaged backgrounds. These findings align with previous research showing that younger individuals tend to have more sensitive circadian rhythms (Liao, Shi, Li, & Han, 2025), and that socioeconomic hardship can amplify the physiological and psychological effects of sleep disruption, leading to increased mental health risks (Etindele, Faustin, Holmes, & Weinstein, 2021). These results suggest that improving sleep regularity may be an effective and low-cost strategy to mitigate depression and anxiety risks in high-risk groups, particularly younger populations and socioeconomically disadvantaged individuals.

The association between sleep regularity and mental health outcomes, persisting after stratification by sleep duration, is particularly noteworthy. This relationship parallels findings by Zheng et al. and Yiallourou et al., who observed that sleep regularity and duration were all important factors associated with health conditions (Zheng et al., 2024), and sleep regularity displayed a U-shaped association with risk of incident dementia, operating through mechanisms distinct from sleep duration alone (Yiallourou et al., 2024). Collectively, these findings highlight that maintaining regular sleep–wake cycles may be equally or potentially more important than achieving recommended sleep duration.

Several plausible biological mechanisms may explain the observed associations between sleep regularity and mental health outcomes. First, irregular sleep patterns can disrupt the synchronization of central and peripheral circadian clocks (Charrier et al., 2017), potentially altering the expression of clock genes that regulate various physiological processes. This desynchronization may affect neurotransmitter systems implicated in mood regulation, including serotonin, dopamine, and norepinephrine (Vadnie & McClung, 2017). Second, disrupted sleep–wake cycles may impair hypothalamic–pituitary–adrenal (HPA) axis function, leading to dysregulated cortisol secretion patterns (Koch et al., 2017). Chronic HPA axis dysfunction has been consistently linked to both depression and anxiety disorders (Gilbert et al., 2017). Third, irregular sleep may compromise prefrontal cortex function and emotion regulation capabilities. Neuroimaging studies have shown that even acute sleep disruption can enhance amygdala reactivity to negative stimuli while reducing functional connectivity with prefrontal regions that modulate emotional responses (Goldstein & Walker, 2014; Krause et al., 2017). Fourth, emerging evidence suggests that sleep irregularity may promote systemic inflammation, as evidenced by elevated levels of pro-inflammatory cytokines observed in individuals with disrupted circadian rhythms (Wright et al., 2015). This is particularly relevant as chronic low-grade inflammation is increasingly recognized as a potential pathophysiological pathway to mood disorders (Miller & Raison, 2016). Additionally, a recent work by Logan and McClung further elucidates how circadian disturbances often precede and potentially contribute to mood disorders, suggesting a causal pathway from sleep irregularity to psychiatric symptoms (Logan & McClung, 2019).

Our findings have important implications for clinical practice and public health strategies. For public health, our findings underscore the importance of education about sleep regularity as a modifiable risk factor and could provide additional mental health benefits at the population level. Current sleep guidelines primarily emphasize duration. Our results suggest that these guidelines should be expanded to incorporate recommendations for maintaining regular sleep–wake patterns, similar to recent calls by the National Sleep Foundation’s consensus statement on sleep regularity (Sletten et al., 2023). From a clinical perspective, healthcare providers should routinely assess sleep regularity alongside other sleep parameters when evaluating patients’ sleep health, particularly those at risk of or experiencing depression and anxiety (Sun et al., 2025). Simple interventions to improve sleep regularity, such as maintaining consistent bedtimes and wake times, may represent an underutilized approach to mental health prevention and management.

Our study has strengths that enhance the reliability and significance of the findings. The use of wrist-worn accelerometers to objectively measure sleep patterns eliminated the recall bias inherent in self-reported sleep data, providing a more accurate assessment of sleep regularity and duration under free-living conditions. The prospective design, with a large sample size and extended follow-up period, allowed for a robust temporal analysis of the relationship between sleep parameters and subsequent mental health outcomes.

Despite these strengths, several limitations warrant consideration when interpreting our results. First, we acknowledge several important limitations regarding causal inference. Despite our prospective design and multiple sensitivity analyses, the observational nature of our study precludes definitive causal conclusions. Although we excluded incident cases occurring up to 7 years post-baseline and adjusted for subclinical symptoms, we cannot completely rule out reverse causality given the long prodromal phases of depression and anxiety. Furthermore, despite extensive covariate adjustment, including polygenic risk scores and personality traits, residual confounding from unmeasured factors remains possible. Second, the accelerometer-based assessment of sleep had inherent limitations. The algorithm we used to determine sleep and wake relied on identifying a main sleep period time window and did not account for napping, which may contribute to overall sleep patterns. Our sleep regularity assessment was based on a single 7-day measurement period, which may not fully capture long-term sleep patterns or changes over time. Additionally, the accelerometer provides an estimate of sleep based on an algorithm, which may not perfectly align with polysomnography, the gold standard for sleep measurement (Boulos et al., 2019). Third, the predominantly White ethnic composition of the cohort restricts the applicability of results to more diverse populations. Fourth, depression and anxiety diagnoses identified through medical records may underestimate the true prevalence of these conditions, as many individuals with mental health disorders do not seek professional help. Although we supplemented this with questionnaire-based assessments in a sensitivity analysis, some cases may still have been missed.

## Conclusion

In conclusion, our study provides compelling evidence that regular sleep patterns are associated with a reduced risk of depression and anxiety. Even among individuals who achieve the recommended sleep duration, consistency in sleep–wake timing remains an independent and significant factor. These results underscore the importance of placing equal emphasis on sleep regularity and duration in depression and anxiety prevention and management.

## Supporting information

Li et al. supplementary material Li et al. supplementary material

## Data Availability

Data used in this study are available on application to the UK Biobank (www.ukbiobank.ac.uk). Analytical methods and study materials will be available to other researchers from the corresponding authors upon reasonable request for the purposes of reproducing the results or replicating the procedure.

## Abbreviations

BMI: body mass index
CI: confidence interval
GAD: generalized anxiety disorder
HR: hazard ratio
PHQ: patient health questionnaire
RCS: restricted cubic spline
SRI: sleep regularity index

## Supplementary material

The supplementary material for this article can be found at http://doi.org/10.1017/S0033291725101281.
