# Supplementary material for: Regular sleep patterns, not just duration, critical for mental health: association of accelerometer-derived sleep regularity with incident depression and anxiety
Source: Psychol Med. 2025 Aug 15;55:e239. doi: 10.1017/S0033291725101281 (PMC12404321; doi:10.1017/S0033291725101281)
Supplement: Li et al. supplementary material [file S0033291725101281sup001.docx]

**Supplementary materials**

**Supplementary Method**

Supplementary Method 1. Construction of PRSs for depression and anxiety.

Supplementary Method 2. Questionnaire screening of depression and anxiety.

**Supplementary Figure**

Supplementary Figure 1. Flow chart of the study design.

**Supplementary Tables**

Supplementary Table 1. Assessment of Healthy Diet Score in this study.

Supplementary Table 2. The definition of variables in this study.

Supplementary Table 3. The comparison between excluded population and final study cohort results.

Supplementary Table 4. Subgroup analyses for the association between SRI and depression risk.

Supplementary Table 5. Subgroup analyses for the association between SRI and anxiety risk.

Supplementary Table 6. Sensitivity analyses for excluding participants with missing or unknown covariates.

Supplementary Table 7. Sensitivity analyses for further adjusting for potential confounders.

Supplementary Table 8. Sensitivity analyses for adding PHQ-9 and GAD-7 to define depression and anxiety.

Supplementary Table 9. Sensitivity analyses for excluding depression and anxiety cases that occurred within the first two, five, and seven years of follow-up.

Supplementary Table 10. Sensitivity analyses for stricter baseline exclusion criteria with PHQ-4 positive and psychotropic medication use.

Supplementary Table 11. The combined effects of SRI and sleep duration with depression risk.

Supplementary Table 12. The combined effects of SRI and sleep duration with anxiety risk.

Supplementary Table 13. Association between SRI and depression risk according to sleep duration stratification.

Supplementary Table 14. Association between SRI and anxiety risk according to sleep duration stratification.

**Supplementary Method 1. Construction of PRSs for depression and anxiety.**

The procedure for genotyping and imputation of single-nucleotide polymorphisms (SNPs) used in the UK Biobank study has been described elsewhere in more detail.(Bycroft et al., 2018) We calculated PRSs of depression (Wray et al., 2018) and anxiety (Friligkou et al., 2024) based on independent SNPs identified from large, previously published genome-wide association studies (GWAS).

For each relevant GWAS, we reviewed the original manuscripts and supplementary materials to extract variants meeting the following criteria: (i) genome-wide significance (*P* < 5×10⁻⁸), (ii) minor allele frequency (MAF) ≥ 0.01 in European populations, and (iii) availability of effect size estimates (odds ratios, ORs). When multiple GWAS were available for the same trait, we prioritized the largest study by sample size for effect size extraction (Bianco et al., 2021; Choi, Jia, Wen, Long, & Zheng, 2020; Fernandez-Rozadilla et al., 2023; Gharahkhani et al., 2016; Sharma, Tapper, Collins, & Hamady, 2022). For variants reported across multiple studies, we required consistent direction of association and replication at *P* < 0.05.

In cases where multiple correlated variants were reported for the same locus, we selected the SNPs with the smallest reported *P*-value using the linkage disequilibrium clumping procedure (at r² < 0.2) in PLINK. For variants not available in the UK Biobank genotyping data, we included strongly correlated SNPs (r² > 0.8) as proxies. SNPs with allele mismatches or MAF differences greater than 0.10 compared to the European population data from the 1000 Genomes Project were excluded.

We computed PRSs as weighted sums of risk alleles using the following formula: PRS = Σ(βᵢ × SNPᵢ), where βᵢ represents the natural logarithm of the OR for SNP i, and SNPᵢ represents the imputed allele. PRSs were subsequently standardized to have a mean of 0 and standard deviation of 1 within the analytical sample.

**Supplementary Method 2.** **Questionnaire screening of depression and anxiety.**

At baseline, depression and anxiety symptoms were assessed only by Patient Health Questionnaire (PHQ)-4 questionnaire (Stanhope, 2016). Participants were required to rate, on a four-point Likert scale from 0 (not at all) to 3 (nearly every day), their response to four items: a) “frequency of depressed mood in the last 2 weeks” (UK Biobank Data-Field: 2050), b) “frequency of unenthusiasm/disinterest in the last 2 weeks” (UK Biobank Data-Field: 2060), c) “frequency of tenseness/restlessness in the last 2 weeks” (UK Biobank Data-Field: 2070), and d) “frequency of tiredness/lethargy in the last 2 weeks” (UK Biobank Data-Field: 2080). Total score ranged from 0 to 12, and a score of was considered emotional disorder positive. A total score of for items 1 and 2 was considered as positive for depression, and a total score of for items 3 and 4 was considered as positive for anxiety based on the reported criteria (Stanhope, 2016).

During the survey, mental health status was assessed using PHQ-9 (an updated PHQ-4)(Kroenke, Spitzer, & Williams, 2001) and Generalized Anxiety Disorder (GAD)-7 questionnaires(Spitzer, Kroenke, Williams, & Löwe, 2006) between 2016 and 2017 with the same four-point Likert scale from 0 (not at all) to 3 (nearly every day). PHQ-9 consists of 9 items for thoughts and feelings in the last 2 week: *a*) “recent thoughts of suicide or self-harm” (Suicidal ideation; UK Biobank Data-Field: 20513), *b*) “trouble falling or staying asleep, or sleeping too much” (Sleeping problems; UK Biobank Data-Field: 20517), *c*) “recent changes in speed/amount of moving or speaking” (Psychomotor changes; UK Biobank Data-Field: 20518), *d*) “recent feelings of inadequacy” (Feelings of inadequacy; UK Biobank Data-Field: 20507). *e*) “recent feelings of tiredness or low energy” (Fatigue; UK Biobank Data-Field: 20519), *f*) “recent feelings of depression” (Depressed mood; UK Biobank Data-Field: 20510), *g*) “recent trouble concentrating on things” (Cognitive problems; UK Biobank Data-Field: 20508), *h*) “recent poor appetite or overeating” (Appetite changes; UK Biobank Data-Field: 20511), and *i*) “recent lack of interest or pleasure in doing things” (Anhedonia; UK Biobank Data-Field: 20514). GAD-7 consists of seven items for thoughts and feelings in the last 2 week: *a*) “recent inability to stop or control worrying” (Worrying control; UK Biobank Data-Field: 20509), *b*) “recent restlessness” (Restlessness; UK Biobank Data-Field: 20516), *c*) “recent trouble relaxing” (Lack of relaxation; UK Biobank Data-Field: 20515), *d*) “recent easy annoyance or irritability” (Irritability; UK Biobank Data-Field: 20505), *e*) “recent worrying too much about different things” (Generalized worrying; UK Biobank Data-Field: 20520), *f*) “recent feelings of foreboding” (Foreboding; UK Biobank Data-Field: 20512), and *g*) “recent feelings of nervousness or anxiety” (Anxiety feeling; UK Biobank Data-Field: 20506). Any item with a score of was considered as positive for this symptom. A PHQ-9 or GAD-7 total score of was considered as depression or anxiety symptoms positive according to the corresponding criteria(Kroenke et al., 2001; Spitzer et al., 2006).

**Reference**

Bianco, C., Jamialahmadi, O., Pelusi, S., Baselli, G., Dongiovanni, P., Zanoni, I., . . . Valenti, L. (2021). Non-invasive stratification of hepatocellular carcinoma risk in non-alcoholic fatty liver using polygenic risk scores. *J Hepatol, 74*(4), 775-782. doi:10.1016/j.jhep.2020.11.024

Bycroft, C., Freeman, C., Petkova, D., Band, G., Elliott, L. T., Sharp, K., . . . Marchini, J. (2018). The UK Biobank resource with deep phenotyping and genomic data. *Nature, 562*(7726), 203-209. doi:10.1038/s41586-018-0579-z

Choi, J., Jia, G., Wen, W., Long, J., & Zheng, W. (2020). Evaluating polygenic risk scores in assessing risk of nine solid and hematologic cancers in European descendants. *Int J Cancer, 147*(12), 3416-3423. doi:10.1002/ijc.33176

Fernandez-Rozadilla, C., Timofeeva, M., Chen, Z., Law, P., Thomas, M., Schmit, S., . . . Peters, U. (2023). Deciphering colorectal cancer genetics through multi-omic analysis of 100,204 cases and 154,587 controls of European and east Asian ancestries. *Nat Genet, 55*(1), 89-99. doi:10.1038/s41588-022-01222-9

Friligkou, E., Løkhammer, S., Cabrera-Mendoza, B., Shen, J., He, J., Deiana, G., . . . Polimanti, R. (2024). Gene discovery and biological insights into anxiety disorders from a large-scale multi-ancestry genome-wide association study. *Nat Genet, 56*(10), 2036-2045. doi:10.1038/s41588-024-01908-2

Gharahkhani, P., Fitzgerald, R. C., Vaughan, T. L., Palles, C., Gockel, I., Tomlinson, I., . . . Schumacher, J. (2016). Genome-wide association studies in oesophageal adenocarcinoma and Barrett's oesophagus: a large-scale meta-analysis. *Lancet Oncol, 17*(10), 1363-1373. doi:10.1016/S1470-2045(16)30240-6

Kroenke, K., Spitzer, R. L., & Williams, J. B. (2001). The PHQ-9: validity of a brief depression severity measure. *J Gen Intern Med, 16*(9), 606-613. doi:10.1046/j.1525-1497.2001.016009606.x

Sharma, S., Tapper, W. J., Collins, A., & Hamady, Z. Z. R. (2022). Predicting Pancreatic Cancer in the UK Biobank Cohort Using Polygenic Risk Scores and Diabetes Mellitus. *Gastroenterology, 162*(6), 1665-1674 e1662. doi:10.1053/j.gastro.2022.01.016

Spitzer, R. L., Kroenke, K., Williams, J. B., & Löwe, B. (2006). A brief measure for assessing generalized anxiety disorder: the GAD-7. *Arch Intern Med, 166*(10), 1092-1097. doi:10.1001/archinte.166.10.1092

Stanhope, J. (2016). Patient Health Questionnaire-4. *Occup Med (Lond), 66*(9), 760-761. doi:10.1093/occmed/kqw165

Wray, N. R., Ripke, S., Mattheisen, M., Trzaskowski, M., Byrne, E. M., Abdellaoui, A., . . . Sullivan, P. F. (2018). Genome-wide association analyses identify 44 risk variants and refine the genetic architecture of major depression. *Nat Genet, 50*(5), 668-681. doi:10.1038/s41588-018-0090-3

**Supplementary Figure 1. Flow chart of the study design.**

| **Diet Component** | **Intake Goal** | **Field IDs** |
| --- | --- | --- |
| Fruits | ≥ 3 servings/day | 1309: “About how many pieces of FRESH fruit would you eat per DAY?”  1319: “About how many pieces of DRIED fruit would you eat per DAY?” |
| Vegetables | ≥ 3 servings/day | 1289: “On average how many heaped tablespoons of COOKED vegetables would you eat per DAY?”  1299: “On average how many heaped tablespoons of SALAD or RAW vegetables would you eat per DAY?” |
| Whole grains | ≥ 3 servings/day | 1438: “How many slices of bread do you eat each WEEK?”  1448: “What type of bread do you mainly eat?”  1458: “How many bowls of cereal do you eat a WEEK?"  1468: "What type of cereal do you mainly eat?” |
| Fish | ≥ 2 servings/week | 1329: “How often do you eat oily fish? (e.g. sardines, salmon, mackerel, herring)”  1339: “How often do you eat other types of fish? (e.g. cod, tinned tuna, haddock)” |
| Refined grains | ≤ 1.5 servings/day | 1438: “How many slices of bread do you eat each WEEK?"  1448: "What type of bread do you mainly eat?”  1458: “How many bowls of cereal do you eat a WEEK?"  1468: "What type of cereal do you mainly eat?” |
| Processed meats | ≤ 1 serving/week | 1349: “How often do you eat processed meats (such as bacon, ham, sausages, meat pies, kebabs, burgers, chicken nuggets)”  3680: “How old were you when you last ate any kind of meat? (Enter "0" if you have never eaten meat in your lifetime)” |
| Red meats | ≤ 1.5 serving/week | 1359: “How often do you eat chicken, turkey or other poultry? (Do not count processed meats)”  1369: “How often do you eat beef? (Do not count processed meats)”  1379: “How often do you eat lamb/mutton? (Do not count processed meats)”  1389: “How often do you eat pork? (Do not count processed meats such as bacon or ham)”  3680: “How old were you when you last ate any kind of meat? (Enter "0" if you have never eaten meat in your lifetime)” |

**Supplementary Table 1. Assessment of Healthy Diet Score in this study.**

We calculated the diet quality score by evaluating the consumption of fruits, vegetables, processed meat, red meat, fish, whole grains, and refined grains. A point was awarded for each of the following criteria: the average intake of fruits ≥ 3 servings/day; vegetables ≥ 3 servings/day; fish ≥ 2 servings/week; processed meat ≤ 1 servings/week; red meat ≤ 1.5 servings/week; whole grain ≥ 3 servings/week; and refined grain ≤ 1.5 servings/week. As a result, the diet quality score ranged from 0 to 7, with higher scores reflecting better diet quality, and ≥ 4 was considered a healthy diet.

**Supplementary Table 2. The definition of variables in this study.**

| **Variables** | **Field IDs** | **Definitions of variables** |
| --- | --- | --- |
| **Outcomes** | | |
| Depression | 41270  130894  130896 | ICD 10 code: F32, F33. |
| Anxiety | 41270  130904  130906  130908  130910  130912  130914  130916 | ICD 10 code: F40–F48. |
| Lost follow-up | 191 | Date lost to follow-up. |
| **Exposures** | | |
| SRI | 90187 | Raw accelerometer statistics. |
| Sleep duration | 90187 | Raw accelerometer statistics. |
| **Covariates** | | |
| Age at recruitment | 21022 | Age was treated as a continuous variable. |
| Sex | 31 | Data-Coding in the UK biobank: male and female. |
| Ethnic background | 21000 | Data-Coding in the UK biobank: White, mixed, Asian, black, Chinese, and others ethnic group. We combined white as a group, and others as a group. |
| Body mass index | 21001 | Body mass index was treated as a continuous variable. |
| Education levels | 6138 | Data-Coding in the UK biobank: “College or university degree”, “A level / AS levels or equivalent”, “O levels / GCSEs or equivalent”, “CSEs or equivalent”, “NVQ or HND or HNC or equivalent”, “Other professional qualifications”, “None of the above”, “Prefer not to answer”. We combined “College or university degree” into a group, “A level / AS levels or equivalent” and “O levels / GCSEs or equivalent” and “CSEs or equivalent” and “NVQ or HND or HNC or equivalent” and “Other professional qualifications” into a group, “None of the above” into a group, and created an “unknown/missing” response category for “Prefer not to answer” or missing data. |
| Annual household income | 738 | Data-Coding in the UK biobank: “(Less than £18,000)”, “(£18,000 to £30,999)”, “(£31,000 to £51,999)”, “(£52,000 to £100,000)”, “(Greater than £100,000)”, “Do not know”, “Prefer not to answer”. We combined “(Less than £18,000)” and “(£18,000 to £30,999)” into a group, “(£31,000 to £51,999)”, “(£52,000 to £100,000)”, and “(Greater than £100,000)” into a group and created an “unknown/missing” group response category for “Do not know”, “Prefer not to answer”, or missing data. |
| Townsend deprivation index | 22189 | Townsend deprivation index calculated immediately prior to participant joining UK Biobank. Based on the preceding national census output areas. Each participant is assigned a score corresponding to the output area in which their postcode is located. Townsend deprivation index was treated as a continuous variable and missing value with the average fill. |
| Smoking status | 20116 | Data-Coding in the UK biobank: “Never”, “Previous”, “Current”, “Prefer not to answer”. We created an “unknown/missing” response category for “Prefer not to answer” or missing data. |
| Alcohol status | 20117 | Data-Coding in the UK biobank: “Never”, “Previous”, “Current”, “Prefer not to answer”. We created an “unknown/missing” response category for “Prefer not to answer” or missing data. |
| Physical activity | 22040 | Total Metabolic Equivalent Task minutes Each week for all activity including walking, moderate and vigorous activity. |
| Employment shift | 826 | Data-Coding in the UK biobank: “Never/rarely”, “Sometimes”, “Usually”, “Always”, “Do not know”, “Prefer not to answer”. We combined “Never/rarely” into a group, “Sometimes”, “Usually”, and “Always” into a group, and created an “unknown/missing” group response category for “Do not know”, “Prefer not to answer”, or missing data. |
| Season of accelerometer wear | 90003 | The date and time that the recording was programmed to start. |

Abbreviations: A, advanced; CSE, Certificate of Secondary Education; GCSE, General Certificate of Secondary Education; HNC, Higher National Certificate; HND, Higher National Diploma; NVQ, National Vocational Qualification; O, ordinary.

**Supplementary Table 3. The comparison between excluded population and final study cohort results.**

| **Characteristics** | **Final study cohort**  **(N=79,666)** | **Excluded population**  **(N=24,017)** | **Total**  **(N=103,683)** |
| --- | --- | --- | --- |
| **Age, year** | 61.53 ± (7.87) | 60.36 ± (7.83) | 61.26 ± (7.87) |
| **BMI, kg/m^2^** | 26.62 ± (4.43) | 27.12 ± (4.86) | 26.73 ± (4.54) |
| **Townsend deprivation index** | -1.77 ± (2.79) | -1.52 ± (2.93) | -1.71 ± (2.83) |
| **Sex** | | | |
| Female | 43,620 (54.75) | 14,664 (61.07) | 58,242 (56.21) |
| Male | 36,046 (45.25) | 9,353 (38.94) | 45,369 (43.79) |
| **Ethnic background** | | | |
| White | 77,124 (96.81) | 23,246 (96.79) | 100,370 (96.81) |
| Non-white ^a^ | 2,542 (3.19) | 771 (3.21) | 3,313 (3.19) |
| **Annual household income, £** | | | |
| < 31,000 | 26,921 (33.78) | 9,013 (37.53) | 35,934 (34.66) |
| ≥ 31,000 | 44,499 (55.86) | 12,483 (51.98) | 56,982 (54.96) |
| Unknown | 8,246 (10.36) | 2,521 (10.49) | 10,767 (10.38) |
| **Education status** | | | |
| High | 34,475 (43.27) | 10,126 (42.16) | 44,601 (43.02) |
| Middle | 37,803 (47.43) | 11,703 (48.73) | 49,506 (47.75) |
| No above | 6,592 (8.27) | 1,868 (7.78) | 8,460 (8.16) |
| Unknown | 796 (1.03) | 320 (1.33) | 1,116 (1.07) |
| **Smoking status** | | | |
| never | 45,905 (57.62) | 13,023 (54.22) | 58,928 (56.84) |
| current | 5,147 (6.46) | 2,124 (8.84) | 7,271 (7.01) |
| previous | 28,401 (35.65) | 8,734 (36.37) | 37,135 (35.82) |
| Unknown | 213 (0.27) | 136 (0.57) | 349 (0.33) |
| **Alcohol consumption status** | | | |
| never | 2,265 (2.84) | 736 (3.06) | 3,001 (2.89) |
| current | 75,393 (94.64) | 22,266 (92.71) | 97,659 (94.19) |
| previous | 1,935 (2.43) | 920 (3.83) | 2,855 (2.75) |
| Unknown | 73 (0.09) | 95 (0.40) | 168 (0.17) |
| **Physical activity level ^b^** | | | |
| Low | 11,011 (13.82) | 3,741 (15.58) | 14,752 (14.23) |
| Moderate | 49,306 (61.89) | 14,650 (60.99) | 63,956 (61.68) |
| High | 19,349 (24.29) | 5,626 (23.43) | 24,975 (24.09) |
| **Diet quality ^c^** | | | |
| Healthy | 53,281 (66.88) | 15,961 (66.46) | 69,242 (66.78) |
| Unhealthy | 26,385 (33.12) | 8,056 (33.54) | 34,441 (33.22) |
| **Employment shift** | | | |
| no | 42,915 (53.87) | 12,795 (53.28) | 55,710 (53.731) |
| yes | 6,289 (7.89) | 2,233 (9.30) | 8,522 (8.219) |
| Unknown | 30,462 (38.24) | 8,989 (37.42) | 39,451 (38.05) |
| **Season of accelerometer wear** | | | |
| Spring | 18,059 (22.67) | 5,532 (23.034) | 23,591 (22.753) |
| Summer | 20,688 (25.97) | 6,742 (28.072) | 27,430 (26.456) |
| Autumn | 23,814 (29.89) | 6,962 (28.988) | 30,776 (29.683) |
| Winter | 17,105 (21.47) | 4,781 (19.907) | 21,886 (21.109) |

Abbreviations: BMI, body mass index; MET, metabolic equivalent of task.

Data are presented as mean ± standard deviation for continuous variables and n (%) for categorical variables.

^a^ The ethnic background of non-White includes mixed, Asian, Black, Chinese, and other ethnic group.

^b^ Physical activity was divided into three levels according to metabolic equivalent tasks (MET): low (< 600 MET minutes/week), medium (600-3,000 MET minutes/week), and high (> 3,000 MET minutes/week)

^c^ Diet score was calculated based on the consumption of fruits, vegetables, processed meat, red meat, fish, whole grains, and refined grains. The diet score ranged from 0 to 7, with higher scores reflecting better diet quality, and ≥ 4 was considered a healthy diet.

**Supplementary Table 4. Subgroup analyses for the association between SRI and depression risk.**

| **Subgroups** | **Cases/Total** | **Sleep Regularity Index ^a^** | | | ***P* for interaction** |
| --- | --- | --- | --- | --- | --- |
|  |  | **Irregular**  **(Q_1_)** | **Moderate Irregular**  **(Q_2_-Q_4_)** | **Regular**  **(Q_5_)** |  |
| **Age, years** |  |  |  |  | 0.002 |
| < 60 | 669/30,148 | 1.00 (Ref.) | 0.78 (0.65-0.93) | 0.47 (0.36-0.62) |  |
| ≥ 60 | 977/49,518 | 1.00 (Ref.) | 0.80 (0.69-0.93) | 0.71 (0.58-0.88) |  |
| **Sex** |  |  |  |  | 0.208 |
| Female | 1,037/43,620 | 1.00 (Ref.) | 0.78 (0.66-0.91) | 0.56 (0.46-0.69) |  |
| Male | 609/36,046 | 1.00 (Ref.) | 0.82 (0.69-0.98) | 0.77 (0.58-1.03) |  |
| **Body mass index ^b^** |  |  |  |  | 0.109 |
| Normal | 557/31,699 | 1.00 (Ref.) | 0.84 (0.67-1.05) | 0.55 (0.41-0.73) |  |
| Overweight | 655/33,119 | 1.00 (Ref.) | 0.76 (0.63-0.91) | 0.60 (0.46-0.78) |  |
| Obesity | 434/14,848 | 1.00 (Ref.) | 0.80 (0.65-0.99) | 0.84 (0.61-1.16) |  |
| **Annual household income, £** |  |  |  |  | 0.804 |
| < 31,000 | 719/26,921 | 1.00 (Ref.) | 0.79 (0.67-0.95) | 0.62 (0.49-0.80) |  |
| ≥ 31,000 | 710/44,499 | 1.00 (Ref.) | 0.82 (0.68-0.99) | 0.60 (0.47-0.78) |  |
| **Diet quality** ^c^ |  |  |  |  | 0.562 |
| Healthy | 1,073/53,281 | 1.00 (Ref.) | 0.77 (0.67-0.90) | 0.63 (0.51-0.77) |  |
| Unhealthy | 573/26,385 | 1.00 (Ref.) | 0.85 (0.70-1.03) | 0.59 (0.44-0.79) |  |
| **Education level** |  |  |  |  | 0.657 |
| High | 600/34,475 | 1.00 (Ref.) | 0.78 (0.64-0.95) | 0.52 (0.40-0.69) |  |
| Medium | 836/37,803 | 1.00 (Ref.) | 0.80 (0.67-0.94) | 0.68 (0.55-0.85) |  |
| No above | 179/6,592 | 1.00 (Ref.) | 0.91 (0.64-1.28) | 0.70 (0.41-1.19) |  |
| **Townsend deprivation index** |  |  |  |  | 0.171 |
| < Median | 729/39,759 | 1.00 (Ref.) | 0.84 (0.69-1.02) | 0.71 (0.55-0.90) |  |
| ≥ Median | 917/39,907 | 1.00 (Ref.) | 0.78 (0.67-0.91) | 0.55 (0.44-0.69) |  |
| **Physical activity level ^d^** |  |  |  |  | 0.763 |
| Low | 265/11,011 | 1.00 (Ref.) | 0.85 (0.64-1.12) | 0.64 (0.42-0.97) |  |
| Medium | 988/49,306 | 1.00 (Ref.) | 0.77 (0.66-0.89) | 0.57 (0.46-0.71) |  |
| High | 393/19,349 | 1.00 (Ref.) | 0.86 (0.67-1.10) | 0.71 (0.51-0.99) |  |

Abbreviations: CI, confidence interval; HR, hazard ratio; MET, metabolic equivalent tasks; Q, quantile; Ref, reference; SRI, Sleep Regularity Index.

^a^ HRs and 95% CIs were calculated using Cox regression models that were adjusted for age at recruitment, sex, body mass index, ethnicity, annual household income, education level, smoking status, Townsend deprivation index, alcohol consumption status, physical activity level, healthy diet score, employment shift, season of accelerometer wear, and sleep duration, unless a certain variable was the basis of the stratification.

^b^ Body mass index was divided into three levels: normal (< 25 kg/m^2^), overweight (25-30 kg/m^2^), and obesity (≥ 30 kg/m^2^).

^c^ Diet score was calculated based on the consumption of fruits, vegetables, processed meat, red meat, fish, whole grains, and refined grains. The diet score ranged from 0 to 7, with higher scores reflecting better diet quality, and ≥ 4 was considered a healthy diet.

^d^ Physical activity was divided into three levels according to MET: low (< 600 MET minutes/week), medium (600-3,000 MET minutes/week), and high (> 3,000 MET minutes/week).

**Supplementary Table 5. Subgroup analyses for the association between SRI and anxiety risk.**

| **Subgroups** | **Cases/Total** | **Sleep Regularity Index ^a^** | | | ***P* for interaction** |
| --- | --- | --- | --- | --- | --- |
|  |  | **Irregular**  **(Q_1_)** | **Moderate Irregular**  **(Q_2_-Q_4_)** | **Regular**  **(Q_5_)** |  |
| **Age, years** |  |  |  |  | 0.383 |
| < 60 | 803/30,148 | 1.00 (Ref.) | 0.78 (0.65-0.92) | 0.69 (0.55-0.86) |  |
| ≥ 60 | 1,294/49,518 | 1.00 (Ref.) | 0.83 (0.72-0.95) | 0.64 (0.53-0.77) |  |
| **Sex** |  |  |  |  | 0.990 |
| Female | 1,387/43,620 | 1.00 (Ref.) | 0.80 (0.70-0.93) | 0.66 (0.55-0.78) |  |
| Male | 710/36,046 | 1.00 (Ref.) | 0.84 (0.71-0.99) | 0.68 (0.52-0.89) |  |
| **Body mass index ^b^** |  |  |  |  | 0.582 |
| Normal | 808/31,699 | 1.00 (Ref.) | 0.76 (0.63-0.92) | 0.61 (0.49-0.77) |  |
| Overweight | 842/33,119 | 1.00 (Ref.) | 0.79 (0.67-0.94) | 0.62 (0.50-0.78) |  |
| Obesity | 447/14,848 | 1.00 (Ref.) | 0.92 (0.74-1.14) | 0.88 (0.64-1.22) |  |
| **Annual household income, £** |  |  |  |  | 0.782 |
| < 31,000 | 865/26,921 | 1.00 (Ref.) | 0.80 (0.68-0.94) | 0.59 (0.47-0.74) |  |
| ≥ 31,000 | 938/44,499 | 1.00 (Ref.) | 0.84 (0.71-1.00) | 0.73 (0.59-0.91) |  |
| **Diet quality ^c^** |  |  |  |  | 0.970 |
| Healthy | 1,420/53,281 | 1.00 (Ref.) | 0.82 (0.71-0.93) | 0.66 (0.56-0.79) |  |
| Unhealthy | 677/26,385 | 1.00 (Ref.) | 0.82 (0.68-0.98) | 0.69 (0.53-0.89) |  |
| **Education level** |  |  |  |  | 0.063 |
| High | 729/34475 | 1.00 (Ref.) | 0.79 (0.66-0.94) | 0.55 (0.43-0.71) |  |
| Medium | 1,084/37,803 | 1.00 (Ref.) | 0.79 (0.68-0.92) | 0.72 (0.59-0.88) |  |
| No above | 236/6592 | 1.00 (Ref.) | 1.02 (0.74-1.41) | 0.88 (0.57-1.37) |  |
| **Townsend deprivation index** |  |  |  |  | 0.002 |
| < Median | 967/39759 | 1.00 (Ref.) | 0.95 (0.80-1.13) | 0.85 (0.69-1.05) |  |
| ≥ Median | 1,130/39907 | 1.00 (Ref.) | 0.75 (0.66-0.86) | 0.55 (0.45-0.67) |  |
| **Physical activity level ^d^** |  |  |  |  | 0.088 |
| Low | 303/11011 | 1.00 (Ref.) | 0.98 (0.74-1.29) | 0.65 (0.44-0.97) |  |
| Medium | 1,306/49306 | 1.00 (Ref.) | 0.75 (0.65-0.86) | 0.59 (0.50-0.71) |  |
| High | 488/19349 | 1.00 (Ref.) | 0.93 (0.73-1.17) | 0.89 (0.67-1.19) |  |

Abbreviations: CI, confidence interval; HR, hazard ratio; MET, metabolic equivalent tasks; Q, quantile; Ref, reference.

^a^ HRs and 95% CIs were calculated using Cox regression models that were adjusted for age at recruitment, sex, body mass index, ethnicity, annual household income, education level, smoking status, Townsend deprivation index, alcohol consumption status, physical activity level, healthy diet score, employment shift, season of accelerometer wear, and sleep duration, unless a certain variable was the basis of the stratification.

^b^ Body mass index was divided into three levels: normal (< 25 kg/m^2^), overweight (25-30 kg/m^2^), and obesity (≥ 30 kg/m^2^).

^c^ Diet score was calculated based on the consumption of fruits, vegetables, processed meat, red meat, fish, whole grains, and refined grains. The diet score ranged from 0 to 7, with higher scores reflecting better diet quality, and ≥ 4 was considered a healthy diet.

^d^ Physical activity was divided into three levels according to MET: low (< 600 MET minutes/week), medium (600-3,000 MET minutes/week), and high (> 3,000 MET minutes/week).

**Supplementary Table 6. Sensitivity analyses for excluding participants with missing or unknown covariates**

| **SRI** | **Cases/Total** | **HR (95% CI)** | |
| --- | --- | --- | --- |
|  |  | **Model 1 ^a^** | **Model 2 ^b^** |
| **Depression** | | | |
| Irregular (Q_1_) | 222/9,686 | 1.00 (Ref.) | 1.00 (Ref.) |
| Moderate Irregular (Q_2_-Q_4_) | 536/29,058 | 0.79 (0.68-0.93) | 0.85 (0.73-1.00) |
| Regular (Q_5_) | 131/9,685 | 0.57 (0.45-0.71) | 0.62 (0.50-0.78) |
| *P* for trend |  | < 0.001 | < 0.001 |
| Per SD increment |  | 0.85 (0.79-0.90) | 0.88 (0.82-0.94) |
| **Anxiety** | | | |
| Irregular (Q_1_) | 256/9,686 | 1.00 (Ref.) | 1.00 (Ref.) |
| Moderate Irregular (Q_2_-Q_4_) | 676/29,058 | 0.83 (0.72-0.96) | 0.87 (0.75-1.01) |
| Regular (Q_5_) | 220/9,685 | 0.78 (0.65-0.94) | 0.82 (0.68-0.99) |
| *P* for trend |  | 0.006 | 0.033 |
| Per SD increment |  | 0.91 (0.85-0.96) | 0.93 (0.87-0.98) |

Abbreviations: CI, confidence interval; HR, hazard ratio; Q, quantile; Ref, reference; SD, standard deviation.

^a^ Model 1 was adjusted for age at recruitment, sex, body mass index, and ethnicity.

^b^ Model 2 was further adjusted for annual household income, education level, smoking status, Townsend deprivation index, alcohol consumption status, physical activity level, healthy diet score, employment shift, season of accelerometer wear, and sleep duration based on model 1.

**Supplementary Table 7. Sensitivity analyses for further adjusting for potential confounders.**

| **Outcomes** | **Cases/Total** | **HR (95% CI)** | |
| --- | --- | --- | --- |
|  |  | **Model 1 ^a^** | **Model 2 ^b^** |
| **Depression** | | | |
| Irregular (Q_1_) | 310/12,276 | 1.00 (Ref.) | 1.00 (Ref.) |
| Moderate Irregular (Q_2_-Q_4_) | 702/38,009 | 0.72 (0.63-0.83) | 0.80 (0.70-0.92) |
| Regular (Q_5_) | 190/12,844 | 0.57 (0.47-0.69) | 0.67 (0.55-0.80) |
| *P* for trend |  | < 0.001 | < 0.001 |
| Per SD increment |  | 0.82 (0.78-0.87) | 0.87 (0.83-0.92) |
| **Anxiety** | | | |
| Irregular (Q_1_) | 362/12,276 | 1.00 (Ref.) | 1.00 (Ref.) |
| Moderate Irregular (Q_2_-Q_4_) | 928/38,009 | 0.78 (0.69-0.88) | 0.84 (0.74-0.95) |
| Regular (Q_5_) | 279/12,844 | 0.66 (0.56-0.77) | 0.74 (0.63-0.87) |
| *P* for trend |  | < 0.001 | < 0.001 |
| Per SD increment |  | 0.87 (0.83-0.91) | 0.91 (0.86-0.96) |

Abbreviations: CI, confidence interval; HR, hazard ratio; Q, quantile; Ref, reference; SD, standard deviation.

^a^ Model 1 was adjusted for age at recruitment, sex, body mass index, and ethnicity.

^b^ Model 2 was further adjusted for annual household income, education level, smoking status, Townsend deprivation index, alcohol consumption status, physical activity level, healthy diet score, employment shift, season of accelerometer wear, sleep duration, polygenic risk scores for depression or anxiety, region, chronotype, neuroticism scores, baseline diabetes, baseline hypertension, and baseline hyperlipidemia based on model 1.

**Supplementary Table 8. Sensitivity analyses for adding PHQ-9 and GAD-7 to define depression and anxiety.**

| **Outcomes** | **Cases/Total** | **HR (95% CI)** | |
| --- | --- | --- | --- |
|  |  | **Model 1 ^a^** | **Model 2 ^b^** |
| **Depression** | | | |
| Irregular (Q_1_) | 1,038/15,934 | 1.00 (Ref.) | 1.00 (Ref.) |
| Moderate Irregular (Q_2_-Q_4_) | 2,090/47,799 | 0.66 (0.61-0.71) | 0.72 (0.67-0.78) |
| Regular (Q_5_) | 517/15,933 | 0.48 (0.43-0.54) | 0.54 (0.49-0.61) |
| *P* for trend |  | < 0.001 | < 0.001 |
| Per SD increment |  | 0.77 (0.75-0.79) | 0.81 (0.79-0.84) |
| **Anxiety** | | | |
| Irregular (Q_1_) | 871/15,934 | 1.00 (Ref.) | 1.00 (Ref.) |
| Moderate Irregular (Q_2_-Q_4_) | 2,132/47,799 | 0.74 (0.69-0.81) | 0.80 (0.73-0.86) |
| Regular (Q_5_) | 633/15,933 | 0.62 (0.56-0.69) | 0.68 (0.61-0.76) |
| *P* for trend |  | < 0.001 | < 0.001 |
| Per SD increment |  | 0.85 (0.82-0.88) | 0.88 (0.85-0.91) |

Abbreviations: CI, confidence interval; HR, hazard ratio; Q, quantile; Ref, reference; SD, standard deviation.

^a^ Model 1 was adjusted for age at recruitment, sex, body mass index, and ethnicity.

^b^ Model 2 was further adjusted for annual household income, education level, smoking status, Townsend deprivation index, alcohol consumption status, physical activity level, healthy diet score, employment shift, season of accelerometer wear, and sleep duration based on model 1.

**Supplementary Table 9. Sensitivity analyses for excluding depression and anxiety cases that occurred within the first two, five, and seven years of follow-up.**

| **Outcomes** | **Cases/Total** | **HR (95% CI)** | |
| --- | --- | --- | --- |
|  |  | **Model 1 ^a^** | **Model 2 ^b^** |
| **Depression** | | | |
| **Two years follow-up** | | | |
| Irregular (Q_1_) | 330/15,830 | 1.00 (Ref.) | 1.00 (Ref.) |
| Moderate Irregular (Q_2_-Q_4_) | 764/47,598 | 0.76 (0.66-0.86) | 0.83 (0.73-0.95) |
| Regular (Q_5_) | 192/15,878 | 0.56 (0.47-0.68) | 0.63 (0.53-0.76) |
| *P* for trend |  | < 0.001 | < 0.001 |
| Per SD increment |  | 0.82 (0.78-0.87) | 0.86 (0.82-0.91) |
| **Five years follow-up** | | | |
| Irregular (Q_1_) | 169/15,669 | 1.00 (Ref.) | 1.00 (Ref.) |
| Moderate Irregular (Q_2_-Q_4_) | 374/47,208 | 0.73 (0.60-0.88) | 0.79 (0.66-0.96) |
| Regular (Q_5_) | 96/15,782 | 0.56 (0.43-0.73) | 0.63 (0.48-0.82) |
| *P* for trend |  | < 0.001 | < 0.001 |
| Per SD increment |  | 0.81 (0.75-0.88) | 0.85 (0.79-0.92) |
| **Seven years follow-up** | | | |
| Irregular (Q_1_) | 112/15,612 | 1.00 (Ref.) | 1.00 (Ref.) |
| Moderate Irregular (Q_2_-Q_4_) | 250/47,084 | 0.73 (0.58-0.91) | 0.80 (0.64-1.01) |
| Regular (Q_5_) | 67/15,753 | 0.59 (0.43-0.80) | 0.67 (0.49-0.91) |
| *P* for trend |  | < 0.001 | 0. 01 |
| Per SD increment |  | 0.80 (0.73-0.88) | 0.85 (0.77-0.93) |
| **Anxiety** | | | |
| **Two years follow-up** | | | |
| Irregular (Q_1_) | 387/15,817 | 1.00 (Ref.) | 1.00 (Ref.) |
| Moderate Irregular (Q_2_-Q_4_) | 987/47,544 | 0.79 (0.70-0.89) | 0.84 (0.74-0.95) |
| Regular (Q_5_) | 274/15,856 | 0.62 (0.53-0.73) | 0.68 (0.57-0.79) |
| *P* for trend |  | < 0.001 | < 0.001 |
| Per SD increment |  | 0.86 (0.82-0.90) | 0.89 (0.85-0.94) |
| **Five years follow-up** | | | |
| Irregular (Q_1_) | 207/15,637 | 1.00 (Ref.) | 1.00 (Ref.) |
| Moderate Irregular (Q_2_-Q_4_) | 513/47,070 | 0.78 (0.66-0.92) | 0.84 (0.71-0.99) |
| Regular (Q_5_) | 138/15,720 | 0.61 (0.49-0.76) | 0.67 (0.54-0.84) |
| *P* for trend |  | < 0.001 | < 0.001 |
| Per SD increment |  | 0.86 (0.80-0.92) | 0.89 (0.84-0.96) |
| **Seven years follow-up** | | | |
| Irregular (Q_1_) | 141/15,571 | 1.00 (Ref.) | 1.00 (Ref.) |
| Moderate Irregular (Q_2_-Q_4_) | 355/46,912 | 0.79 (0.65-0.96) | 0.86 (0.70-1.05) |
| Regular (Q_5_) | 98/15,680 | 0.64 (0.49-0.83) | 0.71 (0.54-0.92) |
| *P* for trend |  | < 0.001 | 0. 012 |
| Per SD increment |  | 0.85 (0.79-0.93) | 0.89 (0.82-0.97) |

Abbreviations: CI, confidence interval; HR, hazard ratio; Q, quantile; Ref, reference; SD, standard deviation.

^a^ Model 1 was adjusted for age at recruitment, sex, body mass index, and ethnicity.

^b^ Model 2 was further adjusted for annual household income, education level, smoking status, Townsend deprivation index, alcohol consumption status, physical activity level, healthy diet score, employment shift, season of accelerometer wear, and sleep duration based on model 1.

**Supplementary Table 10. Sensitivity analyses for stricter baseline exclusion criteria with PHQ-4 positive and psychotropic medication use.**

| **Outcomes** | **Cases/Total** | **HR (95% CI)** | |
| --- | --- | --- | --- |
|  |  | **Model 1 ^a^** | **Model 2 ^b^** |
| **Depression** | | | |
| Irregular (Q_1_) | 207/13,427 | 1.00 (Ref.) | 1.00 (Ref.) |
| Moderate Irregular (Q_2_-Q_4_) | 510/41,535 | 0.80 (0.67-0.94) | 0.86 (0.73-1.01) |
| Regular (Q_5_) | 122/14,047 | 0.56 (0.45-0.71) | 0.62 (0.49-0.78) |
| *P* for trend |  | < 0.001 | < 0.001 |
| Per SD increment |  | 0.84 (0.79-0.90) | 0.873 (0.82-0.93) |
| **Anxiety** | | | |
| Irregular (Q_1_) | 328/13,427 | 1.00 (Ref.) | 1.00 (Ref.) |
| Moderate Irregular (Q_2_-Q_4_) | 850/41,535 | 0.79 (0.70-0.90) | 0.84 (0.74-0.96) |
| Regular (Q_5_) | 234/14,047 | 0.62 (0.52-0.74) | 0.67 (0.56-0.79) |
| *P* for trend |  | < 0.001 | < 0.001 |
| Per SD increment |  | 0.87 (0.83-0.92) | 0.90 (0.85-0.95) |

Abbreviations: CI, confidence interval; HR, hazard ratio; Q, quantile; Ref, reference; SD, standard deviation.

^a^ Model 1 was adjusted for age at recruitment, sex, body mass index, and ethnicity.

^b^ Model 2 was further adjusted for annual household income, education level, smoking status, Townsend deprivation index, alcohol consumption status, physical activity level, healthy diet score, employment shift, season of accelerometer wear, and sleep duration based on model 1.

**Supplementary Table 11. The combined effects of SRI and sleep duration with depression risk.**

| **Sleep duration** | **Cases/Total** | **HR (95% CI)** | |
| --- | --- | --- | --- |
|  |  | **Model 1 ^a^** | **Model 2 ^b^** |
| **Regular (Q_5_)** | | | |
| Meeting guidelines | 152/10,404 | 1.00 (Ref.) | 1.00 (Ref.) |
| Not meeting guidelines | 95/5,529 | 1.18 (0.91-1.53) | 1.15 (0.89-1.49) |
| **Moderate Irregular (Q_2_-Q_4_)** | | | |
| Meeting guidelines | 534/27,930 | 1.34 (1.12-1.60) | 1.31 (1.09-1.57) |
| Not meeting guidelines | 431/19,869 | 1.52 (1.26-1.83) | 1.45 (1.21-1.75) |
| **Irregular (Q_1_)** | | | |
| Meeting guidelines | 165/7,197 | 1.67 (1.33-2.08) | 1.52 (1.21-1.90) |
| Not meeting guidelines | 269/8,737 | 2.19 (1.79-2.69) | 1.91 (1.55-2.35) |
| ***P* for trend** |  | < 0.001 | < 0.001 |

Abbreviations: CI, confidence interval; HR, hazard ratio; Q, quantile; Ref, reference; SRI, Sleep Regularity Index.

^a^ Model 1 was adjusted for age at recruitment, sex, body mass index, and ethnicity.

^b^ Model 2 was further adjusted for annual household income, education level, smoking status, Townsend deprivation index, alcohol consumption status, physical activity level, healthy diet score, employment shift, and season of accelerometer wear, based on model 1.

**Supplementary Table 12. The combined effects of SRI and sleep duration with anxiety risk.**

| **Sleep duration** | **Cases/Total** | **HR (95% CI)** | |
| --- | --- | --- | --- |
|  |  | **Model 1 ^a^** | **Model 2 ^b^** |
| **Regular (Q_5_)** | | | |
| Meeting guidelines | 228/10,404 | 1.00 (Ref.) | 1.00 (Ref.) |
| Not meeting guidelines | 123/5,529 | 1.02 (0.82-1.27) | 1.01 (0.81-1.26) |
| **Moderate Irregular (Q_2_-Q_4_)** | | | |
| Meeting guidelines | 700/27,930 | 1.21 (1.04-1.40) | 1.18 (1.02-1.38) |
| Not meeting guidelines | 542/19,869 | 1.33 (1.13-1.55) | 1.29 (1.10-1.50) |
| **Irregular (Q_1_)** | | | |
| Meeting guidelines | 204/7,197 | 1.48 (1.22-1.79) | 1.37 (1.13-1.66) |
| Not meeting guidelines | 300/8,737 | 1.78 (1.49-2.13) | 1.61 (1.35-1.93) |
| ***P* for trend** |  | < 0.001 | < 0.001 |

Abbreviations: CI, confidence interval; HR, hazard ratio; Q, quantile; Ref, reference; SRI, Sleep Regularity Index.

^a^ Model 1 was adjusted for age at recruitment, sex, body mass index, and ethnicity.

^b^ Model 2 was further adjusted for annual household income, education level, smoking status, Townsend deprivation index, alcohol consumption status, physical activity level, healthy diet score, employment shift, and season of accelerometer wear, based on model 1.

**Supplementary Table 13. Association between SRI and depression risk according to sleep duration stratification.**

| **SRI** | **Cases/Total** | **HR (95% CI)** | | ***P* for interaction** |
| --- | --- | --- | --- | --- |
|  |  | **Model 1 ^a^** | **Model 2 ^b^** |  |
| **Meeting guidelines** | | | | 0.57 |
| Regular (Q_5_) | 152/10,404 | 1.00 (Ref.) | 1.00 (Ref.) |  |
| Moderate Irregular (Q_2_-Q_4_) | 534/27,930 | 1.34 (1.12-1.61) | 1.30 (1.08-1.56) |  |
| Irregular (Q_1_) | 165/7,197 | 1.67 (1.33-2.09) | 1.48 (1.18-1.86) |  |
| *P* for trend |  | < 0.001 | 0.001 |  |
| Per SD increment |  | 0.85 (0.80-0.91) | 0.89 (0.83-0.95) |  |
| **Not meeting guidelines** | | | |  |
| Regular (Q_5_) | 95/5,529 | 1.00 (Ref.) | 1.00 (Ref.) |  |
| Moderate Irregular (Q_2_-Q_4_) | 431/19,869 | 1.29 (1.03-1.61) | 1.26 (1.01-1.58) |  |
| Irregular (Q_1_) | 269/8,737 | 1.85 (1.46-2.36) | 1.69 (1.33-2.16) |  |
| *P* for trend |  | < 0.001 | < 0.001 |  |
| Per SD increment |  | 0.80 (0.75-0.85) | 0.83 (0.78-0.89) |  |

Abbreviations: CI, confidence interval; HR, hazard ratio; Q, quantile; Ref, reference; SD, standard deviation; SRI, Sleep Regularity Index.

^a^ Model 1 was adjusted for age at recruitment, sex, body mass index, and ethnicity.

^b^ Model 2 was further adjusted for annual household income, education level, smoking status, Townsend deprivation index, alcohol consumption status, physical activity level, healthy diet score, employment shift, and season of accelerometer wear, based on model 1.

**Supplementary Table 14. Association between SRI and anxiety risk according to sleep duration stratification.**

| **SRI** | **Cases/Total** | **HR (95% CI)** | | ***P* for interaction** |
| --- | --- | --- | --- | --- |
|  |  | **Model 1 ^a^** | **Model 2 ^b^** |  |
| **Meeting guidelines** | | | | 0.55 |
| Regular (Q_5_) | 228/10,404 | 1.00 (Ref.) | 1.00 (Ref.) |  |
| Moderate Irregular (Q_2_-Q_4_) | 700/27,930 | 1.21 (1.04-1.40) | 1.18 (1.01-1.37) |  |
| Irregular (Q_1_) | 204/7,197 | 1.48 (1.22-1.80) | 1.35 (1.11-1.64) |  |
| *P* for trend |  | < 0.001 | 0.003 |  |
| Per SD increment |  | 0.90 (0.85-0.96) | 0.93 (0.88-0.99) |  |
| **Not meeting guidelines** | | | |  |
| Regular (Q_5_) | 123/5,529 | 1.00 (Ref.) | 1.00 (Ref.) |  |
| Moderate Irregular (Q_2_-Q_4_) | 542/19,869 | 1.29 (1.06-1.58) | 1.28 (1.05-1.55) |  |
| Irregular (Q_1_) | 300/8,737 | 1.74 (1.40-2.16) | 1.62 (1.30-2.02) |  |
| *P* for trend |  | < 0.001 | < 0.001 |  |
| Per SD increment |  | 0.82 (0.77-0.87) | 0.84 (0.79-0.90) |  |

Abbreviations: CI, confidence interval; HR, hazard ratio; Q, quantile; Ref, reference; SD, standard deviation; SRI, Sleep Regularity Index.

^a^ Model 1 was adjusted for age at recruitment, sex, body mass index, and ethnicity.

^b^ Model 2 was further adjusted for annual household income, education level, smoking status, Townsend deprivation index, alcohol consumption status, physical activity level, healthy diet score, employment shift, and season of accelerometer wear, based on model 1
